# Professional Bickerings

**Published:** 1868-09

**Authors:** H. Scott


### Original Communications.

### PROFESSIONAL BICKERINGS.

BY H. SCOTT.

All that I feel, and that my judgment has settled in my mind in regard to professional bickerings and jealousies, I can not ask space to express. I am approaching the close of a somewhat protracted professional career, and feel satisfied with my opinions on this one subject, and speak them as freely as I sincerely believe them.

The aims and ends of the professional Dentist, legitimately stated, are correct judgments on what is to be done, excellence in doing it, merited reputation, and to make money. How are these honorably and certainly to be secured? I will state how I think success is best to be obtained.

There never was a time, perhaps, within the history of Dentistry, when the mania to get money quick and easy approximated the present. In many portions of the country, known to the writer, agents are, to-day, permeating every neighborhood and entering every house to solicit work. They go right to the doors of resident Dentists, enter the families of their patrons, and by the logic of strategy, underbidding, and various artifices not unfrequently get cases to be carried off to another county. Their special business is to extract teeth and prepare mouths, and thus secure, for the office they represent, the making of the artificial dentures. It is needless that I say, to well informed Dentists, that tens of thousands of teeth are thus sacrificed that would easily have been preserved through life by appropriate treatment and filling. Sinful does not express all the wrong, —such practice is criminal.

Dentists meet in convention from remote points and enter into fraternal relations. They freely reciprocate what they have learned and as freely exchange views, and thus good is done. But such men are not competitors, their spheres are too wide apart to allow of jealousy, (generally this is so). But while for a brief time their friendly relations exist, the convention utterly ignores, or does not know, that in many of the towns and small cities through the State or States, where there are from two or three to half a dozen or more resident Dentists, that to a shameful and disgraceful extent these Dentists are not on speaking terms with one another. I want to show the causes and the folly of such a state of things.

An unhealthy desire to get money and reputation is at the bottom of all, but means often defeat ends, and men are slow to find it out, many never see it at all until seeing does no good. A disreputable system of egotistic advertising, as well as over-done "*notices*," is resorted to by some. And then the system of "making friends," and making them on the basis of securing their personal influence and direct interferences in getting cases and extending business generally, defeats itself in more ways than one. It sets others to work to see how many "*friends*" they can secure who will work for them; and thus, in too many instances, needless strife and ill-feeling is gotten up among neighbors about matters that are, after all, none of their business. And so the best strategist sometimes carries the day with less professional merit than his neighbor, who, to-morrow, by a flank movement, or some other movement, in his turn takes and holds the ground for a precarious length of time, for where such a state of things obtains, nothing is secure, and little is rational, and there is no reason to expect that merit will at last triumph over demagogism. And Dentists themselves are to blame for all this and for all the consequences growing out of such a course, and the end of it all can not be otherwise, with the rational, sober, second thought, than a general impairment of the public confidence in the utility of Dentists and their interference with the teeth. And the same is true with all the professions more or less.

Dentists who adopt such a course most sadly mistake their own best interests. I am sure they do. I have made it a special point to try to cultivate the fellowship of Dentists who have settled in my own place, but I have not always succeeded.

Now what I propose, as the result of my most carefully collated thoughts, is this: Let Dentists of the same town or city so manage their own affairs as that their personal friends or the public shall let them quietly alone in their business. Let it be known that they, as a body, repel officious interference with their professional matters. Let them be more friendly among themselves than with outsiders. Let them come together often and tell each other all they know and all they think touching their specialties, and all strive to benefit and help each other. And when this is done all war will cease, and the public appreciation of Dental science will be greatly enlarged. Each man will be before the people on his real deservings, and all will have more friends and make more money. Of the truth of this, I have not one, even feeble doubt. Professional men only publish broadcast their own imperfections and the imperfections of their cause when they quarrel among themselves, and if they can induce their friends to fight for them the chances of war are not improbable.

